# Supplementary material for: Black-necked spitting cobra (Naja nigricollis) phospholipases A2 may cause Trypanosoma brucei death by blocking endocytosis through the flagellar pocket
Source: Sci Rep. 2022 Apr 16;12:6394. doi: 10.1038/s41598-022-10091-5 (PMC9013370; doi:10.1038/s41598-022-10091-5)
Supplement: Supplementary file 1 — Supplementary Figure S1. [file 41598_2022_10091_MOESM1_ESM.pdf]

A

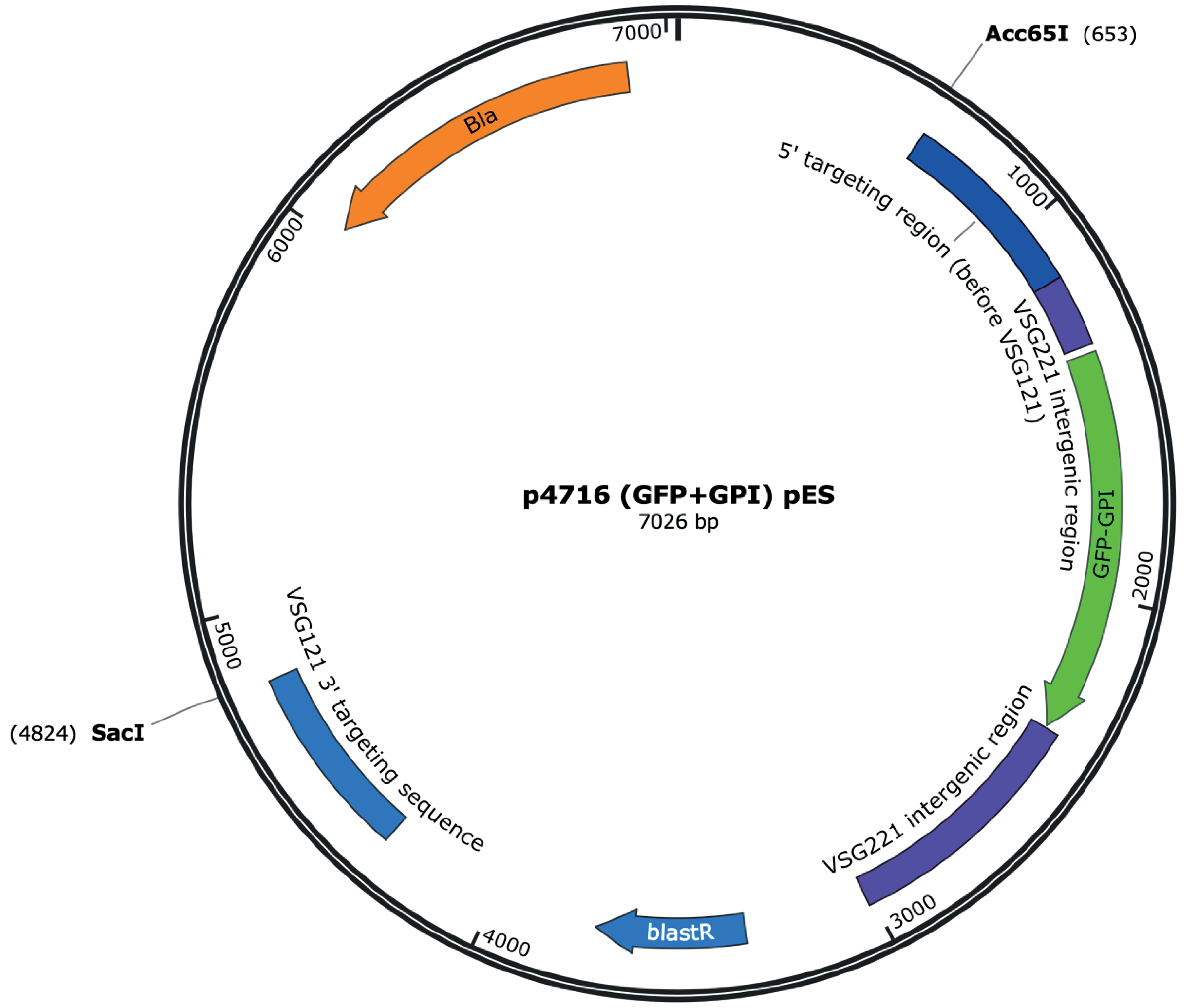

B

Acc65I

GGTACCCTGTTCTAAACATAAACATAGCAATAAATATTATCGGAAACGAGCAAATACAGGAAGGTTGTAATTGAAATAGAGTCACTAAATTATCCGGTAAACCAGTGGAAGTAATAGTAAAA
TTAACAAAAGTATAGAATGAAATAATGTGACTGTCAACAACTTCCGCAGAACAAGGGAAAATAATACATGAAGAGGAAGAGGAGATAATGCACCGTATTTCACTCAGAAATGGATATATTA
TTTTGCCGTGGTGGTGGCGTCTATGTTTCGTTGGCTGATGACGGATTCTAATGTTCAGTTGTTGACAATAAGCAACTCCAAAGCACACAATGGTAAACAAAGGAAATGGAACAAAAGG
AAAGTGCTTGAAAAGATTGCAGTCCTACAACAAAATACAAAAATTGCAGTAAGAAAACACAGAGCAGCTGTGATCACTGCCTTGACTGAGGATATATTTTTCGGAATCGAAAAACTAATC
GTTTATAAAATTAGAAAAGCACTGACTCGAGCACACAAGCATTCTATACGTAAAAGATCTAGTATATAGGAGCAACGCTCTGCCAAAACATAATGGCAAGACAAACGGCCGTGTTTGCC
GCTGATGCTACAGAACCAGCTTAATTTCCAGAAGACGAAAATTTGCATGTTTTCCCACAATATTTTAATTACTCTTGAAGATTGTAGTTATTCCTACGCGACACGAACGCGGCATGCCAA
GTAACCAGGAGGCACGTCTTTTTCTTGCAGTGCTTGTGCTTGCACAGGTGCTTCCAATTCTTGTGGATAGTGCAGCAGAGAAGGGTGCACGTGAGGTGCACACAAACCAGGACCCA
AGTGGTAGTAACACAACAAGTGGTAGTAGTTCCATGGGTCGTAAGGGCGAGGAGCTGTTCACCGGGGTGGTGCCCATCCTGGTCGAGCTGGACGGCGACGTAAACGGCCACAAGT
TCAGCGTGCGTGGCGAGGGCGAGGGCGATGCCACCAACGGCAAGCTGACCCTGAAGTTCATCTGCACCACCGGCAAGCTGCCCGTGCCCTGGCCCACCCTCGTGACCACCCTG
ACCTACGGCGTGCAGTGCTTCGCACGCTACCCCGACCACATGAAGCAGCACGACTTCTTCAAGTCCGCCATGCCCGAAGGCTACGTCCAGGAGCGCACCATCAGTTTCAAGGACG
ACGGCACATACAAGACCCGCGCCGAGGTGAAGTTCGAGGGCGACACCCTGGTGAACCGCATCGAGCTGAAGGGCATCGACTTCAAGGAGGACGGCAACATCCTGGGGCACAAGC
TGGAGTACAACTTTAACAGCCACAACGTCTATATCACAGCCGACAAGCAGAAGAACGGCATCAAGGCAAACTTCAAGATCCGCCACAACGTGGAGGACGGCAGCGTGCAGCTCGCC
GACCACTACCAGCAGAACACCCCCATCGGCGACGGCCCCGTGCTGCTGCCCGACAACCACTACCTGAGCACCCAGTCCGTGCTGAGCAAAGACCCCAACGAGAAGCGCGATCAC
ATGGTCCTGCTGGAGTTCGTGACCGCCGCCGGGATCACTCACGGCATGGACGAGCTGTACAAGGGCGCCAAGAAGGTGGCAGATGAGACAGCAAAGGATGGTAAGACAGGTAAC
ACAAACACAACAGGTAGTAGTAACAGTTTTGTGATTAGTAAGACACCACTTTGGCTTGCAGTGCTTCTTTTTTAATTAATTTCCCCCCTCAAATTTCCCCCCTCCTTTTAAAATTTTCCTT
GCTACTTGAAAACTTTTTGATATATTTTAACACCAAAACCAGCCGAGATTTTGTGTTCTGTGTTTTGTAAGTTGACTGTCTGATTGTCTAGAAATATTTTCTGGCAACTAAAATTTTTTTCT
TTTTTCCTGTTTTTTTTGTAGGTAGGTAGGAATGGGGGGGGGGGGTAGTTAGGTAGGTTAGTTAGGTTAGTTAGGGGGGTTAGTTAGGGGGGTTAGGCTTAGGATTAGGATTAGACTTAG
GCTTAGGATTAGGATTAGGATTAGGATTAGGGTTAATTTTTTCCTCTTTTTTTTTAACTCACACCTCTATCCTGGATTTTTAATTTTTTTTTTTAGCCATTCGCGGCTCCTTTTTTTTTTTTT
GCGCCAATGTTTAATTTTTTATTGTGTTTTCAATTTTTTTGTCAACCATGCAGCGGCTGTTTTGTTATGCGGACCCTAACCCTCCTCCCCCCCCCCCGCCCGCGCACCTCCATTTTTAA
AAATTTTTTTACCGCGTCCTTCAACCAGAATTTTTTTAAATTTTTTAATTTTTTTTTATTTTCCGTGGTTTTGAATCTTAATTTTTCGGGGGAATTCCTGCAGCCCGGGTAGAAAGTGTGACA
ACGTCGCACCATGTGTAGGTTTTCATTTATGTTCTTTCTTTCTTTTTTTTTGTGAATTTGTTTTCTGTCTCAAATGTTTTTAATTCGCTTGGGACCTATGTTTTTCTTGTTTTTTTGCTCACC
CTTTGTGTAGGAGGCACCCTGTCACGTCTGTGGTTGCGTGTATGCCTTCCTTCCCCTTATTCGCTTCTTCCTGTCGTGTCACACCTCTTTCTCTCTCTCCCTTTCCGCCTTTTCTTTCA
ATCTTGTTTTCTCGACCAGCCCTACTAGAGGAGAAAGAACAGTAACCCTTTCATCAAAGAAAATAGTTCAAACGAATTCATATGCCTTTGTCTCAAGAAGAATCCACCCTCATTGAAAGA
GCAACGGCTACAATCAACAGCATCCCCATCTCTGAAGACTACAGCGTCGCCAGCGCAGCTCTCTCTAGCGACGGCCGCATCTTCACTGGTGTCAATGTATATCATTTTACTGGGGGAC
CTTGTGCAGAACTCGTGGTGCTGGGCACTGCTGCTGCTGCGGCAGCTGGCAACCTGACTTGTATCGTCGCGATCGGAAATGAGAACAGGGGCATCTTGAGCCCCTGCGGACGGTG
CCGACAGGTGCTTCTCGATCTGCATCCTGGGATCAAGGCCATAGTGAAGGACAGTGATGGACAGCCGACGGCAGTTGGGATTCGTGAATTGCTGCCCTCTGGTTATGTGTGGGAGG
GCTAATTCGAATAGACGCGGACGGGGCATTTCCCGTTCGTCATTAGCAGTAGGTAATGAAGATGTTTGTTTCTCGTCCCCTTTCTCCTTCGTCCTTCTGTCATTTTGTTCTTTTGTGTTT
ATGTTTTGTTGTTGTTTTCTTTAATTTTTTTTTTTTCTTCCACGTTTGTGTACATCCGCGCGCCACTCTATTCAGAGAGCCACGGATAGTAGAGGAGGTGGGAAGGGTATATGAGGGACAC
GCGTACCATGATGTGGGATGTATTGGGGTCCCTGTCTGTCCTTACGTGACTATGTATGAACCGTCACGTGTAAGATGAGCTAGTGAGATCAACAGTACAACTCATCAACACGCCTTCTT
CTCGTTAAATGTACACAATCTTGATCCTCCACCTTTATGGGTCCCATTGTTTGCCTCTTCCGCTGTGTGGAGTGCGCCTACACGCACTTCTCACTTCGTAAGTGGTGGTGGCGTAAGTA
TTGCCTAATGTTGACTCTATATTCTCCTCTCCTCACCCCCTCGCGGTGCTGATTTCTGACAGATCTTCAAACACTAGATTAAGCAAAGGACTATTCATCCGTTACTAGTACCACCTGTGC
GACGAAGCTGCAAAAGAATAAAGCGGCACCAAGTAGTTCTAACAAGCTGTAGTGAGACCCAGCTGCACGATAGATTAATTTATTATTTTTTAAATTTTGAGTTTTTTTAAATTTATATAAGT
GTAATTCACCACATTAAAAAGGGGGAAAGAGGACTCAAAATGATATTCTAATTAGCTGAAGGAAACGGTGATGAAAATAGATAATAAACATTCCCCAAAAATATTACCACAACTAACTTCT
TTGTTTTTCTCTTTCATGTTGCTAAACTAGACAACAGCGTTAAGCGATGGCCGTGCACAGAGCCCTAGCGGCGTACGCGATTAGTCTTTACGTTTTACTACCCAGAAAATCGGGAGCA
ACAGACAAAGGCGCGATCAAGTTTGAGACGTGGGAGCCGCTCTGTTTACTGACACAAGACTTCGGTAACCTTTACAACAGAGCGCACAAACTTAATCTCGACATCGACACCTACGTA
ACCGCAGCCCACCGCGGTG**GAGCTC**

SacI

Supplementary Figure 1. A. p4716 (GFP-GPI) pES plasmid map. B. Acc65I/SacI insert used for transfection.
